# *N*-(3-Chloro-4-eth­oxy­benzo­yl)-*N*′-(2-meth­oxy­phen­yl)thio­urea

**DOI:** 10.1107/S1600536810054644

**Published:** 2011-01-12

**Authors:** Jing-Han Hu, Zhong-Yi Luo, Chen-Fei Ding, Xiao-Li Song

**Affiliations:** a College of Chemical and Biological Engineering, Lanzhou Jiaotong University, Lanzhou 730070, People’s Republic of China

## Abstract

In the title compound, C_17_H_17_ClN_2_O_3_S, the central carbonyl­thio­urea unit is nearly planar [maximum atomic deviation = 0.019 (3) Å] and makes dihedral angles of 2.47 (7) and 17.76 (6)° with the terminal benzene rings. An intra­molecular N—H⋯O hydrogen bond occurs. Weak inter­molecular C—H⋯S and C—H⋯Cl hydrogen bonding is observed in the crystal structure.

## Related literature

For applications of thio­urea derivatives, see: Antholine & Taketa (1982 ▶); Schroeder (1955 ▶). For related structures, see: Yusof & Yamin (2004*a* ▶,*b* ▶); Ali *et al.* (2004 ▶). For related acyl­thio­urea derivatives, see: Zhang *et al.* (2003 ▶, 2006 ▶).
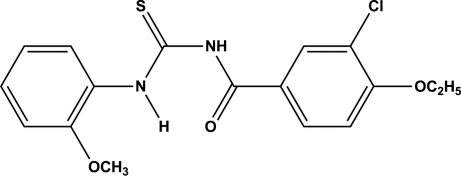


## Experimental

### 

#### Crystal data

C_17_H_17_ClN_2_O_3_S
*M* *_r_* = 364.84
Triclinic, 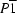

*a* = 7.8238 (8) Å
*b* = 8.4791 (11) Å
*c* = 14.9867 (13) Å
α = 76.365 (7)°
β = 89.384 (5)°
γ = 62.647 (4)°
*V* = 852.65 (16) Å^3^
*Z* = 2
Mo *K*α radiation
μ = 0.36 mm^−1^
*T* = 296 K
0.38 × 0.35 × 0.27 mm

#### Data collection

Bruker APEXII CCD diffractometer
Absorption correction: multi-scan (*SADABS*; Bruker, 2001 ▶) *T*_min_ = 0.874, *T*_max_ = 0.908
4903 measured reflections
3314 independent reflections
2679 reflections with *I* > 2σ(*I*)
*R*_int_ = 0.012

#### Refinement

*R*[*F* ^2^ > 2σ(*F* ^2^)] = 0.061
*wR*(*F* ^2^) = 0.216
*S* = 1.06
3314 reflections
219 parameters
H-atom parameters constrained
Δρ_max_ = 0.33 e Å^−3^
Δρ_min_ = −0.85 e Å^−3^

### 

Data collection: *APEX2* (Bruker, 2007 ▶); cell refinement: *SAINT* (Bruker, 2007 ▶); data reduction: *SAINT*; program(s) used to solve structure: *SHELXTL* (Sheldrick, 2008 ▶); program(s) used to refine structure: *SHELXTL*; molecular graphics: *SHELXTL*; software used to prepare material for publication: *SHELXTL*.

## Supplementary Material

Crystal structure: contains datablocks I, global. DOI: 10.1107/S1600536810054644/xu5125sup1.cif

Structure factors: contains datablocks I. DOI: 10.1107/S1600536810054644/xu5125Isup2.hkl

Additional supplementary materials:  crystallographic information; 3D view; checkCIF report

## Figures and Tables

**Table 1:** Hydrogen-bond geometry (Å, °)

*D*—H⋯*A*	*D*—H	H⋯*A*	*D*⋯*A*	*D*—H⋯*A*
N2—H2⋯O2	0.86	1.88	2.613 (3)	143
C6—H6⋯S1^i^	0.93	2.86	3.468 (2)	124
C14—H14⋯Cl1^ii^	0.93	2.81	3.680 (3)	156

Symmetry codes: (i) 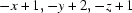; (ii) 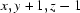.

**Fig. 1.**
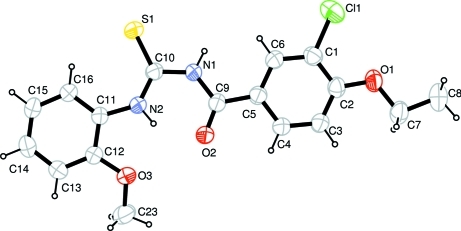
The molecular structure of the title compound, shown with 50% probability displacement ellipsoids.

## supplementary crystallographic information

### Comment

Thiourea derivatives have been studied for their potential use in agriculture, medicine and analytical chemistry (Schroeder, 1955; Antholine & Taketa, 1982). As part of our ongoing work on acylthiourea derivatives (Zhang *et al.*, 2003; Zhang *et al.*, 2006), we present here the structure of the title thiourea derivative, (I).

Benzoylthiourea derivatives can be synthesized from the reaction between benzoylisothiocyanate and amine compounds. In the title compound, (I), the molecular structure and dimensions are similar to those in other benzoylthiourea derivatives, such as *N*-(2-chlorophenyl)-*N*'-(4-methoxybenzoyl)thiourea (Yusof & Yamin, 2004*a*), *N*-(4-methoxybenzoyl-*N*'-(*o*-tolyl)thiourea (Yusof & Yamin, 2004*b*) and *N*-(*p*-methoxybenzoyl)-*N*'-(*o*-methoxyphenyl)thiourea (Ali *et al.*, 2004). The molecule maintains its *trans-cis* configuration with respect to the position of the 3-chloro-4-ethoxybenzoyl and 2-methoxyphenyl groups relative to the S atom across the thiourea C—N bonds.

The central carbonyl thiourea moiety (S1/O2/N1/N2/C9/C10), the 2-methoxyphenyl group (C11–C16/O3/C23) and the 3-chloro-4-ethoxybenzoyl (C1–C6/C8/O1) group are individually planar. The C10—S1, C10—N1 and C10—N2 bond lengths are1.665 (2), 1.392 (2) and 1.333 (3) Å, respectively, comparable with those in *N*-(2-chlorophenyl)-N'- (4-methoxybenzoyl)-thiourea [C═S = 1.662 (2) Å, C8—N1 = 1.386 (3) Å and C8—N2 = 1.331 (3) Å; Yusof & Yamin, 2004*a*] and other benzoylthiourea derivatives. There is one intramolecular hydrogen bonds, *via* N2—H2···O2; as a result, one pseudo-six-membered rings, is formed (Fig. 1).

### Experimental

Potassium thiocyanate (7.5 mmol), 3-chloro-4-ethoxybenzoyl chloride (5 mmol), PEG-400 (3% with respect to ammonium thiocyanate) and dichloromethane (20 ml) were placed in a dried flask and stirred at room temperature for 1 h, then 2-methoxyaniline (5 mmol) was added. The mixture was stirred for 0.5 h at room temperature and a precipitate was formed. This was filtered off, washed with water and dried. yellow single crystals of (I) were obtained from an ethanol–dimethylformamide (1:1) solution.

### Refinement

Methyl H atoms were placed in calculated positions with C—H = 0.96 Å and torsion angles were refined to fit the electron density, *U*_iso_(H) = 1.5*U*_eq_(C). Other H atoms were placed in calculated positions with C—H = 0.93 (aromatic), 0.97 Å (methylene) and N—H = 0.86 Å, and refined in riding mode with U_iso_(H) = 1.2U_eq_(N,C).

### Crystal data

**Table:**

C_17_H_17_ClN_2_O_3_S	*Z* = 2
*M**_r_* = 364.84	*F*(000) = 380
Triclinic, *P*1	*D*_x_ = 1.421 Mg m^−^^3^
Hall symbol: -P 1	Mo *K*α radiation, λ = 0.71073 Å
*a* = 7.8238 (8) Å	Cell parameters from 2087 reflections
*b* = 8.4791 (11) Å	θ = 2.8–29.3°
*c* = 14.9867 (13) Å	µ = 0.36 mm^−^^1^
α = 76.365 (7)°	*T* = 296 K
β = 89.384 (5)°	Block, yellow
γ = 62.647 (4)°	0.38 × 0.35 × 0.27 mm
*V* = 852.65 (16) Å^3^	

### Data collection

**Table:**

Bruker APEXII CCD diffractometer	3314 independent reflections
Radiation source: fine-focus sealed tube	2679 reflections with *I* > 2σ(*I*)
graphite	*R*_int_ = 0.012
φ and ω scans	θ_max_ = 26.0°, θ_min_ = 1.4°
Absorption correction: multi-scan (*SADABS*; Bruker, 2001)	*h* = −9→9
*T*_min_ = 0.874, *T*_max_ = 0.908	*k* = −10→9
4903 measured reflections	*l* = −18→17

### Refinement

**Table:**

Refinement on *F*^2^	Primary atom site location: structure-invariant direct methods
Least-squares matrix: full	Secondary atom site location: difference Fourier map
*R*[*F*^2^ > 2σ(*F*^2^)] = 0.061	Hydrogen site location: inferred from neighbouring sites
*wR*(*F*^2^) = 0.216	H-atom parameters constrained
*S* = 1.06	*w* = 1/[σ^2^(*F*_o_^2^) + (0.1281*P*)^2^ + 0.5121*P*] where *P* = (*F*_o_^2^ + 2*F*_c_^2^)/3
3314 reflections	(Δ/σ)_max_ < 0.001
219 parameters	Δρ_max_ = 0.33 e Å^−^^3^
0 restraints	Δρ_min_ = −0.85 e Å^−^^3^

### Special details

**Table:**

Geometry. All e.s.d.'s (except the e.s.d. in the dihedral angle between two l.s. planes) are estimated using the full covariance matrix. The cell e.s.d.'s are taken into account individually in the estimation of e.s.d.'s in distances, angles and torsion angles; correlations between e.s.d.'s in cell parameters are only used when they are defined by crystal symmetry. An approximate (isotropic) treatment of cell e.s.d.'s is used for estimating e.s.d.'s involving l.s. planes.
Refinement. Refinement of *F*^2^ against ALL reflections. The weighted *R*-factor *wR* and goodness of fit *S* are based on *F*^2^, conventional *R*-factors *R* are based on *F*, with *F* set to zero for negative *F*^2^. The threshold expression of *F*^2^ > σ(*F*^2^) is used only for calculating *R*-factors(gt) *etc*. and is not relevant to the choice of reflections for refinement. *R*-factors based on *F*^2^ are statistically about twice as large as those based on *F*, and *R*- factors based on ALL data will be even larger.

### Fractional atomic coordinates and isotropic or equivalent isotropic displacement parameters (Å^2^)

**Table:**

	*x*	*y*	*z*	*U*_iso_*/*U*_eq_	
C1	0.7479 (5)	0.3322 (4)	0.6563 (2)	0.0500 (7)	
C2	0.9023 (5)	0.1588 (4)	0.6605 (2)	0.0484 (7)	
C3	1.0005 (5)	0.1342 (4)	0.5825 (2)	0.0535 (7)	
H3	1.1037	0.0201	0.5832	0.064*	
C4	0.9454 (5)	0.2778 (4)	0.5048 (2)	0.0507 (7)	
H4	1.0123	0.2591	0.4534	0.061*	
C5	0.7916 (4)	0.4512 (4)	0.50087 (19)	0.0455 (7)	
C6	0.6918 (4)	0.4750 (4)	0.5784 (2)	0.0467 (7)	
H6	0.5868	0.5883	0.5772	0.056*	
C7	1.0853 (5)	−0.1587 (4)	0.7415 (2)	0.0600 (8)	
H7A	1.2106	−0.1668	0.7289	0.072*	
H7B	1.0421	−0.1991	0.6950	0.072*	
C8	1.0993 (6)	−0.2751 (5)	0.8357 (3)	0.0758 (11)	
H8A	1.1418	−0.2333	0.8809	0.114*	
H8B	1.1905	−0.4008	0.8396	0.114*	
H8C	0.9745	−0.2659	0.8472	0.114*	
C9	0.7458 (4)	0.5941 (4)	0.4133 (2)	0.0488 (7)	
C10	0.5447 (4)	0.9307 (4)	0.34023 (19)	0.0435 (6)	
C11	0.5829 (4)	1.0267 (4)	0.17305 (19)	0.0438 (6)	
C12	0.7042 (4)	0.9479 (4)	0.1091 (2)	0.0465 (7)	
C13	0.6871 (5)	1.0502 (4)	0.0204 (2)	0.0557 (8)	
H13	0.7675	0.9962	−0.0217	0.067*	
C14	0.5509 (5)	1.2321 (5)	−0.0058 (2)	0.0607 (8)	
H14	0.5390	1.3018	−0.0657	0.073*	
C15	0.4325 (5)	1.3110 (4)	0.0562 (2)	0.0624 (9)	
H15	0.3413	1.4346	0.0379	0.075*	
C16	0.4464 (5)	1.2103 (4)	0.1452 (2)	0.0552 (8)	
H16	0.3641	1.2656	0.1863	0.066*	
C23	0.9845 (6)	0.6837 (5)	0.0906 (3)	0.0777 (12)	
H23A	0.9323	0.6801	0.0339	0.117*	
H23B	1.0716	0.5608	0.1254	0.117*	
H23C	1.0533	0.7541	0.0770	0.117*	
Cl1	0.6221 (2)	0.36762 (17)	0.75042 (8)	0.0918 (4)	
N1	0.6147 (4)	0.7725 (3)	0.41304 (16)	0.0461 (6)	
H1	0.5705	0.7877	0.4648	0.055*	
N2	0.6167 (4)	0.9049 (3)	0.26084 (16)	0.0480 (6)	
H2	0.6994	0.7928	0.2638	0.058*	
O1	0.9466 (4)	0.0284 (3)	0.74006 (15)	0.0597 (6)	
O2	0.8217 (4)	0.5561 (3)	0.34440 (15)	0.0700 (7)	
O3	0.8333 (4)	0.7658 (3)	0.14238 (16)	0.0636 (7)	
S1	0.38700 (13)	1.12797 (11)	0.36170 (5)	0.0603 (3)	

### Atomic displacement parameters (Å^2^)

**Table:**

	*U*^11^	*U*^22^	*U*^33^	*U*^12^	*U*^13^	*U*^23^
C1	0.0574 (17)	0.0502 (16)	0.0453 (16)	−0.0268 (14)	0.0093 (13)	−0.0143 (13)
C2	0.0594 (18)	0.0439 (15)	0.0419 (15)	−0.0256 (14)	−0.0015 (12)	−0.0080 (12)
C3	0.0589 (18)	0.0428 (15)	0.0447 (16)	−0.0135 (13)	0.0026 (13)	−0.0086 (13)
C4	0.0574 (17)	0.0429 (15)	0.0420 (15)	−0.0150 (13)	0.0073 (12)	−0.0120 (12)
C5	0.0512 (16)	0.0412 (14)	0.0407 (15)	−0.0183 (13)	0.0019 (12)	−0.0115 (12)
C6	0.0514 (16)	0.0394 (14)	0.0470 (16)	−0.0188 (13)	0.0064 (12)	−0.0128 (12)
C7	0.067 (2)	0.0490 (17)	0.0524 (18)	−0.0226 (16)	−0.0052 (15)	−0.0028 (14)
C8	0.087 (3)	0.062 (2)	0.067 (2)	−0.037 (2)	−0.006 (2)	0.0087 (18)
C9	0.0557 (17)	0.0391 (14)	0.0419 (15)	−0.0151 (13)	0.0026 (12)	−0.0086 (12)
C10	0.0443 (15)	0.0400 (14)	0.0414 (14)	−0.0151 (12)	0.0039 (11)	−0.0124 (11)
C11	0.0467 (15)	0.0392 (14)	0.0383 (14)	−0.0154 (12)	0.0033 (11)	−0.0080 (11)
C12	0.0498 (16)	0.0395 (14)	0.0442 (15)	−0.0168 (12)	0.0060 (12)	−0.0091 (12)
C13	0.0631 (19)	0.0518 (17)	0.0456 (16)	−0.0220 (15)	0.0154 (14)	−0.0119 (13)
C14	0.068 (2)	0.0560 (19)	0.0449 (17)	−0.0253 (16)	0.0057 (14)	0.0015 (14)
C15	0.0605 (19)	0.0432 (16)	0.0551 (18)	−0.0080 (14)	0.0061 (15)	0.0023 (14)
C16	0.0524 (17)	0.0445 (16)	0.0475 (16)	−0.0079 (13)	0.0095 (13)	−0.0066 (13)
C23	0.069 (2)	0.053 (2)	0.090 (3)	−0.0121 (17)	0.032 (2)	−0.0168 (19)
Cl1	0.1143 (9)	0.0876 (8)	0.0697 (7)	−0.0445 (7)	0.0403 (6)	−0.0216 (5)
N1	0.0549 (14)	0.0374 (12)	0.0348 (11)	−0.0131 (10)	0.0070 (10)	−0.0083 (9)
N2	0.0556 (14)	0.0361 (12)	0.0380 (12)	−0.0101 (10)	0.0073 (10)	−0.0092 (10)
O1	0.0798 (16)	0.0468 (12)	0.0420 (11)	−0.0253 (11)	0.0027 (10)	−0.0027 (9)
O2	0.0962 (18)	0.0416 (11)	0.0400 (12)	−0.0062 (12)	0.0159 (11)	−0.0103 (9)
O3	0.0711 (15)	0.0417 (11)	0.0552 (13)	−0.0089 (10)	0.0227 (11)	−0.0106 (10)
S1	0.0673 (6)	0.0436 (5)	0.0466 (5)	−0.0055 (4)	0.0105 (4)	−0.0144 (3)

### Geometric parameters (Å, °)

**Table:**

C1—C6	1.371 (4)	C10—N2	1.333 (4)
C1—C2	1.397 (4)	C10—N1	1.392 (4)
C1—Cl1	1.716 (3)	C10—S1	1.665 (3)
C2—O1	1.341 (4)	C11—C16	1.384 (4)
C2—C3	1.396 (4)	C11—C12	1.402 (4)
C3—C4	1.373 (4)	C11—N2	1.408 (4)
C3—H3	0.9300	C12—O3	1.367 (3)
C4—C5	1.395 (4)	C12—C13	1.375 (4)
C4—H4	0.9300	C13—C14	1.372 (5)
C5—C6	1.395 (4)	C13—H13	0.9300
C5—C9	1.477 (4)	C14—C15	1.369 (5)
C6—H6	0.9300	C14—H14	0.9300
C7—O1	1.448 (4)	C15—C16	1.379 (4)
C7—C8	1.492 (5)	C15—H15	0.9300
C7—H7A	0.9700	C16—H16	0.9300
C7—H7B	0.9700	C23—O3	1.402 (4)
C8—H8A	0.9600	C23—H23A	0.9600
C8—H8B	0.9600	C23—H23B	0.9600
C8—H8C	0.9600	C23—H23C	0.9600
C9—O2	1.221 (4)	N1—H1	0.8600
C9—N1	1.383 (4)	N2—H2	0.8600
			
C6—C1—C2	121.7 (3)	N2—C10—S1	127.6 (2)
C6—C1—Cl1	118.8 (2)	N1—C10—S1	117.5 (2)
C2—C1—Cl1	119.4 (2)	C16—C11—C12	118.5 (3)
O1—C2—C3	124.8 (3)	C16—C11—N2	127.1 (3)
O1—C2—C1	117.2 (3)	C12—C11—N2	114.4 (2)
C3—C2—C1	118.0 (3)	O3—C12—C13	124.7 (3)
C4—C3—C2	120.2 (3)	O3—C12—C11	114.4 (2)
C4—C3—H3	119.9	C13—C12—C11	120.9 (3)
C2—C3—H3	119.9	C14—C13—C12	119.7 (3)
C3—C4—C5	121.7 (3)	C14—C13—H13	120.2
C3—C4—H4	119.1	C12—C13—H13	120.2
C5—C4—H4	119.1	C15—C14—C13	120.0 (3)
C6—C5—C4	118.0 (3)	C15—C14—H14	120.0
C6—C5—C9	125.4 (3)	C13—C14—H14	120.0
C4—C5—C9	116.6 (3)	C14—C15—C16	121.1 (3)
C1—C6—C5	120.3 (3)	C14—C15—H15	119.4
C1—C6—H6	119.9	C16—C15—H15	119.4
C5—C6—H6	119.9	C15—C16—C11	119.8 (3)
O1—C7—C8	106.8 (3)	C15—C16—H16	120.1
O1—C7—H7A	110.4	C11—C16—H16	120.1
C8—C7—H7A	110.4	O3—C23—H23A	109.5
O1—C7—H7B	110.4	O3—C23—H23B	109.5
C8—C7—H7B	110.4	H23A—C23—H23B	109.5
H7A—C7—H7B	108.6	O3—C23—H23C	109.5
C7—C8—H8A	109.5	H23A—C23—H23C	109.5
C7—C8—H8B	109.5	H23B—C23—H23C	109.5
H8A—C8—H8B	109.5	C9—N1—C10	128.7 (2)
C7—C8—H8C	109.5	C9—N1—H1	115.6
H8A—C8—H8C	109.5	C10—N1—H1	115.6
H8B—C8—H8C	109.5	C10—N2—C11	132.1 (2)
O2—C9—N1	121.6 (3)	C10—N2—H2	114.0
O2—C9—C5	121.3 (3)	C11—N2—H2	114.0
N1—C9—C5	117.1 (3)	C2—O1—C7	118.0 (2)
N2—C10—N1	114.8 (2)	C12—O3—C23	118.7 (3)
			
C6—C1—C2—O1	179.6 (3)	O3—C12—C13—C14	179.5 (3)
Cl1—C1—C2—O1	−1.3 (4)	C11—C12—C13—C14	0.6 (5)
C6—C1—C2—C3	0.4 (5)	C12—C13—C14—C15	−0.1 (6)
Cl1—C1—C2—C3	179.5 (2)	C13—C14—C15—C16	−0.5 (6)
O1—C2—C3—C4	−178.9 (3)	C14—C15—C16—C11	0.6 (6)
C1—C2—C3—C4	0.2 (5)	C12—C11—C16—C15	−0.1 (5)
C2—C3—C4—C5	0.1 (5)	N2—C11—C16—C15	179.8 (3)
C3—C4—C5—C6	−0.9 (5)	O2—C9—N1—C10	1.6 (5)
C3—C4—C5—C9	−179.8 (3)	C5—C9—N1—C10	−178.8 (3)
C2—C1—C6—C5	−1.3 (5)	N2—C10—N1—C9	−0.9 (5)
Cl1—C1—C6—C5	179.6 (2)	S1—C10—N1—C9	−179.7 (3)
C4—C5—C6—C1	1.5 (5)	N1—C10—N2—C11	179.6 (3)
C9—C5—C6—C1	−179.8 (3)	S1—C10—N2—C11	−1.8 (5)
C6—C5—C9—O2	−169.5 (3)	C16—C11—N2—C10	−6.2 (5)
C4—C5—C9—O2	9.2 (5)	C12—C11—N2—C10	173.6 (3)
C6—C5—C9—N1	11.0 (5)	C3—C2—O1—C7	−10.3 (5)
C4—C5—C9—N1	−170.3 (3)	C1—C2—O1—C7	170.6 (3)
C16—C11—C12—O3	−179.5 (3)	C8—C7—O1—C2	−177.0 (3)
N2—C11—C12—O3	0.7 (4)	C13—C12—O3—C23	13.3 (5)
C16—C11—C12—C13	−0.5 (5)	C11—C12—O3—C23	−167.8 (3)
N2—C11—C12—C13	179.7 (3)		

### Hydrogen-bond geometry (Å, °)

**Table:**

*D*—H···*A*	*D*—H	H···*A*	*D*···*A*	*D*—H···*A*
N2—H2···O2	0.86	1.88	2.613 (3)	143
C6—H6···S1^i^	0.93	2.86	3.468 (2)	124
C14—H14···Cl1^ii^	0.93	2.81	3.680 (3)	156

Symmetry codes: (i) −*x*+1, −*y*+2, −*z*+1; (ii) *x*, *y*+1, *z*−1.
